# Remote Screening for Atrial Fibrillation by a Federal Cardiac Monitoring System in Primary Care Patients in Russia: Results from the Prospective Interventional Multicenter FECAS-AFS Study

**DOI:** 10.5334/gh.1057

**Published:** 2022-01-18

**Authors:** Daria Gognieva, Nelly Vishnyakova, Yulia Mitina, Petr Chomakhidze, Dinara Mesitskaya, Natalia Kuznetsova, Mohamed Khiari, Galina Ryabykina, Sergey Boytsov, Abram Syrkin, Hugo Saner, Philipp Kopylov

**Affiliations:** 1 World-Class Research Center ‘Digital biodesign and personalized healthcare’ I.M. Sechenov First Moscow State Medical University (Sechenov University), Moscow, RU; 2 The state budgetary institution of health care of the Moscow region ‘Mytishchi city clinical hospital’, Mytishchi, RU; 3 Department of Physiology, School of Biomedical Sciences, University of Melbourne, Melbourne, AU; 4 Department of Cardiology, Functional and Ultrasound Diagnostics of N.V. Sklifosovsky Institute for Clinical Medicine, I.M. Sechenov First Moscow State Medical University (Sechenov University), Moscow, RU; 5 Department of new research methods, Federal State budget organization National medical research center of cardiology of the Ministry of healthcare of the Russian Federation, Moscow, RU; 6 ARTORG Center for Biomedical Engineering Research, University of Bern, Bern, CH

**Keywords:** Atrial fibrillation, screening, remote heart rhythm monitoring, single-lead ECG, anticoagulation therapy, stroke

## Abstract

**Background:**

Screening for atrial fibrillation has the potential to significantly reduce cardiovascular morbidity and mortality. However, questions in regard to how to screen, on whom to screen, and the optimal setting of screening remain unanswered.

**Objective:**

To assess the applicability of a federal cardiac monitoring for atrial fibrillation (AF) screening and remote heart rhythm monitoring in patients at high cardiovascular risk in a mixed urban and rural population in Russia.

**Methods:**

This is a prospective multicenter cohort study including 3249 individuals with high cardiovascular risk (mean age 56 ± 12.8 years) from the larger Moscow region who were screened for AF using a smartphone-case based single-lead ECG monitor over a period of 18 month. The endpoints were considered as number of newly diagnosed AF; mean time to diagnosis; number of patients for the first time assigned to anticoagulation therapy; frequency of adverse events.

**Results:**

A trial fibrillation was diagnosed in 126 patients, 36 of them for the first time. The mean time to diagnosis was 3 ± 2 days. Of 36 patients, the CHA2DS2-VASc score was ≥1 in 34 cases, ≥2 in 29 cases. Anticoagulant therapy was first induced in 31 patients. One death in newly diagnosed group and two deaths in chronic group were registered. There were a total of eight hospitalizations: one in newly diagnosed and seven in chronic AF patients.

**Conclusion:**

Our results indicate that a Federal AF screening system in patients at high cardiovascular risk by using a smartphone-case based single lead ECG which is supported by centrally located ECG specialist and central data management is feasible and reliable when performed in a mixed urban and rural area. Further studies are needed to evaluate the full potential of this approach.

## Introduction

The prevalence of atrial fibrillation (AF) in the Russian Federation is high and further increased by 44% from 2010 to 2018. It currently stands at 2536 per 100,000 people (2.5%), the incidence of AF in patients over 70 years being 1.5–5.5. times higher than in patients 50–69 years old [1].

The risk of stroke in patients with AF is about 4.2% per year. Currently, the economic burden of atrial fibrillation in the Russian Federation is estimated to be about 52 billion rubles/year, and a further increase of up to 135 billion rubles/year is expected due to an increasing incidence rate and progress with the availability of invasive diagnostic and therapeutic interventions [1].

We expect that population aging together with an increase in life expectancy amid progress in modern medicine will lead to further elevation of AF patients, with a subsequent reciprocal increase in health care costs [1].

Given the severe consequences of ischemic strokes and a rather high mortality rate, timely screening is considered as the main mechanism to reduce the burden of AF by identification of asymptomatic forms of atrial fibrillation followed by the administration of adequate anticoagulation therapy. However, timely diagnosis of AF is not always possible in particular due to the territorial features of Russia and the inaccessibility. For example, the frequency of outpatient follow-up in patients with chronic heart failure I-III functional class (New York Heart Association Classification of Heart Failure [NYHA]) is once a year, and for patients with VI functional class – two times a year. At the same time such patient has a high risk of developing atrial fibrillation. These patients, especially not in the central regions, cannot always get timely access to medical care. To be consulted by a cardiologist, they must first visit a therapist. The waiting time for both consultations apart can be up to 15 days, and in total become a whole month. At the same time, to register an ECG in 12 leads, the patient also need to stand in line.

The search for measures to improve this situation led to the creation of the Federal Cardiac Monitoring System for AF. This study describes the first results in regard to applicability and results of such a system for AF screening and remote heart rhythm monitoring.

## Materials and Methods

### The structure of the Federal Cardiac Monitoring System

**Figure 1:**
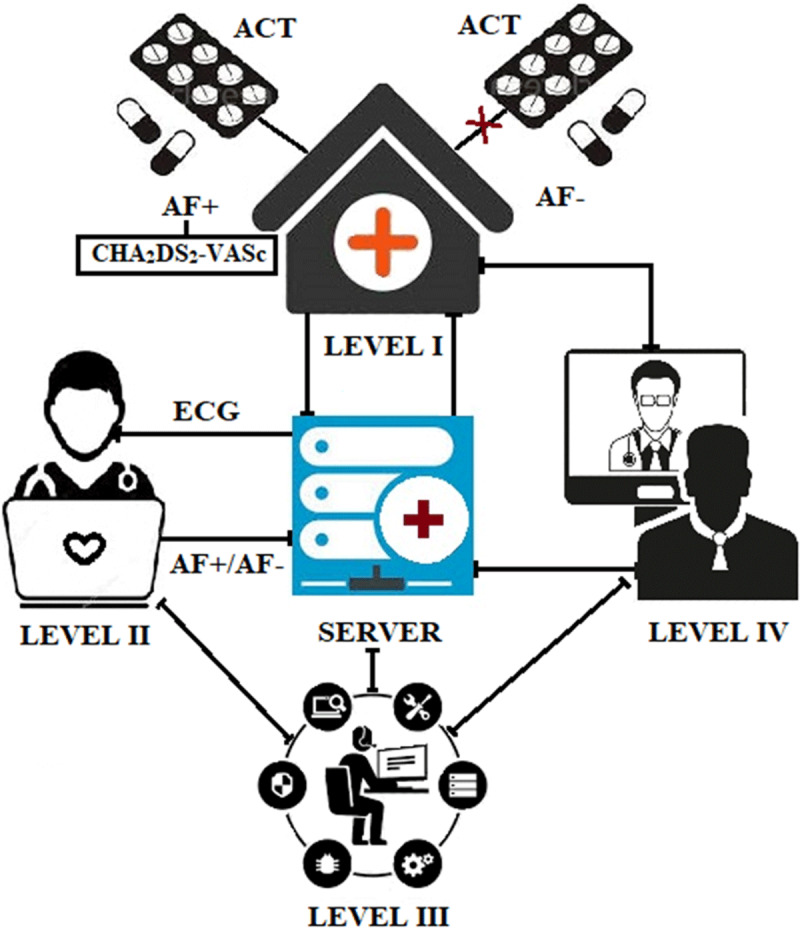
Structure of the Federal Cardiac Monitoring System.

The system includes four main levels (***Figure 1***):

- Level 1: Medical institutions providing direct contact with the patient (three outpatient departments, three affiliates of a city clinical hospital and six rural health posts in the Moscow region);
- Level 2: Expert center (three experts) providing analysis of ECG recordings suspicious for atrial fibrillation;
- Level 3: Technical center (one specialist) providing software and a mobile application to the device;
- Level 4: Administrative center (two specialists) providing the function of regulation and management.

All medical institutions (level 1) were equipped with 25 portable smartphone-case based devices (CardioQVARK®) for recording an ECG in the I standard lead.

### Smartphone-case based ECG device

**Figure 2:**
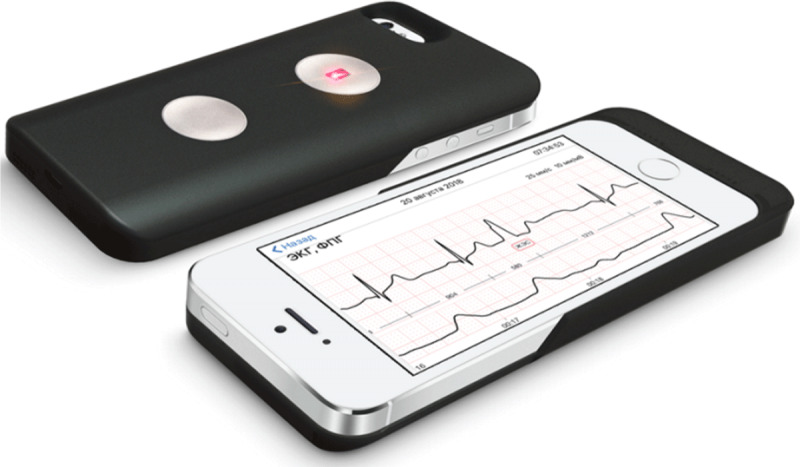
Smartphone-case based ECG device (CardioQVARK®) including also pulse-wave analysis by photopletysmography (not included in this study).

The CardioQVARK® device (registration certificate for the medical device No. RZN 2019/8124 dated February 15, 2019) is a case for iPhone 5, 5s, SE with two electrodes located on the outer surface (***Figure 2***). To record an ECG, a person has place the index fingers on the surface of the electrodes.

The device is also equipped with additional external electrodes for ECG record in II, III, aVR, aVL, aVF, and chest leads if needed. ECG data analysis is performed automatically, including the calculation of the time intervals for RR, P, PR, QRS, QT, QTc, calculation of heart rate variability parameters, and – based on these parameters – identification of recordings suspicious for the presence of an arrhythmia.

### Software

Safe transmission (TLS) and data storage, initial automatic analysis of the received records (calculation of RR, P, PR, QRS, QT, QTc, calculation of arrhythmia and heart rate variability parameters), as well as ensuring interaction between participants, is carried out through API service CardioQVARK®, developed by CardioQVARK LLC.

The software meets the requirements of GOST R IEC 62304-2013 for class A software. To store data in the software, the standard secure isolated storage provided by the iOS operating system is used. Protection against unauthorized access to data is also implemented by using the HTTPS encryption protocol (TLS). To collect, store and send information about the operation of the product, a Bug report system is implemented.

### Study design

Between November 22, 2017, and April 3, 2019 patients from primary care units with high cardiovascular risk were included. However, for practical reasons it was not possible to enroll patients in a strictly consecutive way. Patients were eligible for enrollment if they were ≥20 years of age with one or more of the following risk factors: arterial hypertension; history of ischemic stroke or transient ischemic attacks; type 1 and 2 diabetes; obesity; heart failure or decreased tolerance to physical activity due to dyspnea; coronary artery disease (CAD) or chest pain without established CAD diagnosis; peripheral arterial disease; abnormal heart rhythm (episodes of palpitations, pauses in heartbeat). Patients were excluded if they had acute coronary syndrome; acute ischemic or hemorrhagic stroke; mental illness; a severe concomitant disease with life expectancy less than two years.

Full eligibility criteria and a complete list of endpoints are listed in the trial protocol (NCT04204330 available at *clinicaltrials.gov*).

The trial protocol and all amendments were approved by the appropriate ethics committee.

Doctors at medical facilities with direct contact with the patient acquired informed consent. Further, all patient registered on the website (*https://itunes.apple.com/en/app/cardioqvark/id1320898122*) by themselves, where he/she entered a series of demographic data. After registration the doctor showed the patient how to record an ECG, then the patient recorded ECG on his own for three minutes (five patients with complaints of palpitations who have no AF at the time of the visit received a portable monitor for home monitoring).

Automatic analysis of the recordings was performed, ECG records suspicious for the presence of arrhythmia (n = 126, 3.21%) were sent to analysis in an expert center. The diagnosis of AF was confirmed by experienced cardiologists. Automatically generated protocols in PDF format were sent to the doctors at level 1 of the system for patient management and treatment. To further confirm the diagnosis for patients with newly detected AF, an additional 12-lead ECG or daily ECG monitoring was performed.

**Figure 3:**
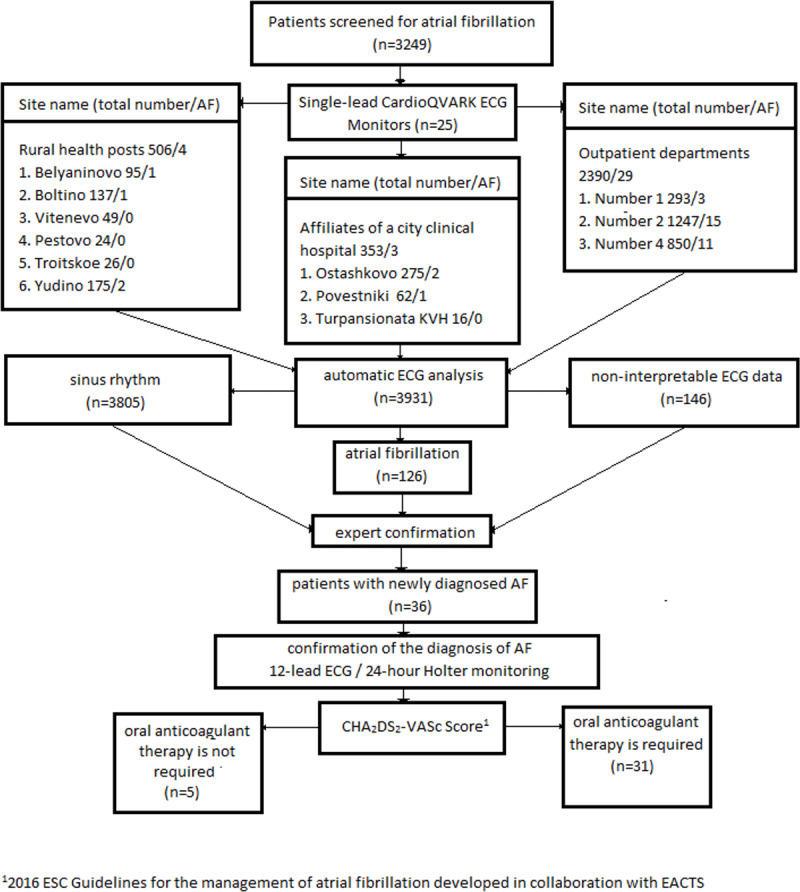
Flow chart of study patients and procedures. AF: atrial fibrillation, ECG: electrocardiogram.

All patients with atrial fibrillation, both previously established and newly identified, were assessed for the degree of risk of thromboembolic complications using the CHA2DS2-VASc score and risk of major bleeding for patients on anticoagulation therapy using the HAS-BLED score. Anticoagulation therapy was prescribed to patients per existing recommendations of the Russian Society of Cardiology and the European Society of Cardiology [2]. The study flow chart is shown in ***Figure 3***.

### Study endpoints

The endpoints were considered as a total number of AF cases newly diagnosed during the study period; the number of patients who, for the first time, were assigned to anticoagulation therapy, mean time to diagnosis; frequency of adverse events.

### Statistical Analysis

Statistical analysis was performed using GraphPad Prism8 (GraphPad Software Inc., USA).

Data are presented as mean ± standard deviation (SD) where normally distributed, as median [interquartile range (IQR)] where not normally distributed, or as a percentage of the group from which they were derived for categorical variables. Normality was tested with the Anderson-Darling (AD) test, where p ≥ 0.05 indicate normal distribution. Parametric testing was performed on data that were normally distributed and non-parametric testing was performed on data that were not normally distributed. A p-value of <0.05 was considered significant for all analyses.

## Results

Between November 22, 2017, and April 3, 2019, a total of 3249 patients from 12 sites were screened, among them 2121.6 (65.28%) women, mean age 56 ± 12.8 years. In 126 patients (3.8%) AF was diagnosed; 36 patients (28%) were newly diagnosed, whereas 90 patients had known chronic AF. Twenty–five patients were lost to follow-up and had to be excluded from further analysis.

Baseline demographic and disease characteristics were as expected for a trial involving patients with AF and were well balanced between groups, except for heart failure (33% in newly diagnosed AF vs 89% in chronic AF, p < 0.0001), coronary artery disease prevalence (39% in newly diagnosed AF vs 90% in chronic AF, p < 0.0001), and HAS-BLED score (2.1 in newly diagnosed AF vs 2.7 in chronic AF, p = 0.0028). Patient characteristics are listed in ***Table 1***.

**Table 1:** Baseline characteristics of the study participants. AF—atrial fibrillation, M—male, F—female, BMI—body mass index, CAD—coronary artery disease, TIA—transitory ischemic attack, CHA_2_DS_2_-VASc Score— the most commonly utilized method to predict thromboembolic risk in atrial fibrillation, HAS-BLED score—a scoring system developed to assess one-year risk of major bleeding in patients taking anticoagulants with atrial fibrillation, SD—standard deviation.


CHARACTERISTICS	ALL SCREENED PATIENTS (N = 3249)	NEWLY DIAGNOSED AF (N = 36)	CHRONIC AF (N = 65)	P-VALUE (NEWLY DIAGNOSED VS. CHRONIC AF)

Sex, M/F, n (%)	1127/2122(34.69/65.28)	19/17 (53/47)	25/41 (38/62)	0.21

Mean age (SD), y	56 y (12.8)	72.7 y (12.4)	71.9 y (11.3)	0.72

Age 65–74 y, n	885	7	13	0.98

Age ≥75 y, n	758	16	33	0.59

BMI (SD), kg/m^2^	32.8 (8.1)	29.6 (5.5)	30.5 (5.3)	0.49

Heart failure, n (%)	203 (6.25)	12 (33)	58 (89)	<0.0001

Hypertension, n (%)	1787 (55)	30 (83)	58 (89)	0.54

Diabetes mellitus, n (%)	358 (11)	6 (17)	7 (11)	0.5361

CAD, n (%)	86 (2.65)	14 (39)	59 (90)	<0.0001

Stroke or TIA in anamnesis, n (%)	9 (0.28)	2 (5.6)	7 (10.8)	0.49

CHA_2_DS_2_-VASc score (SD)	N/A	3.1 (1.7)	3.7 (1.3)	0.06

HAS-BLED score (SD)	N/A	2.1 (1.1)	2.7 (0.9)	0.0028


Mean time to diagnosis was 3 ± 2 days (***Table 2***). Between November 2017 and April 2019 (18 months), one death in newly diagnosed AF group and two deaths in chronic AF group were registered (p = 0.94). There was a total of eight hospitalizations among participants: one (3%) in newly diagnosed AF and seven in chronic AF patients (p = 0.1396).

**Table 2:** Primary and secondary outcome measures in patients with newly diagnosed AF and chronic AF. CHA_2_DS_2_-VASc Score—the most commonly utilized method to predict thromboembolic risk in atrial fibrillation, ACT—anticoagulation therapy, SD—standard deviation. * Thirty–one patients in the newly diagnosed AF group were administered with ACT.


CHARACTERISTICS	NEWLY DIAGNOSED AF (N = 36)	CHRONIC AF (N = 65)	P-VALUE

Time to diagnosis, d (SD)	3 (2)	N/A	N/A

Death, n (%)	1 (3)	2 (3)	0.94

Hospitalization, n (%)	1 (3)	7 (11)	0.1396

Patients with a CHA_2_DS_2_-VASc score ≥ 1, n (%)	34 (94)	65 (100)	0.12

Patients with a CHA_2_DS_2_-VASc score ≥ 2	29 (81)	60 (92)	0.11

Compliance to ACT, n (%)*	20 (64)	57 (88)	0.0127

Not complaint to ACT, n (%)*	7 (22)	3 (5)	0.0118

Ischemic stroke or transient ischemic attack after enrollment, n (%)	0 (0)	0 (0)	>0.9999

Massive hemorrhage after enrollment, n (%)	0 (0)	0 (0)	>0.9999

Hemorrhagic stroke after enrollment, n (%)	0 (0)	0 (0)	>0.9999


Of 36 patients, the CHA_2_DS_2_-VASc score was ≥ 1 in 34 cases, CHA_2_DS_2_-VASc score ≥ 2 in 29 cases. Anticoagulant therapy (ACT) was first induced in 31 (86.11%) patients. Six months after inclusion in the study, patients did not develop ischemic stroke or transient ischemic attack, hemorrhagic stroke. The mean time to diagnosis was 3 ± 2 days (***Table 2***).

Of 65 patients, the CHA_2_DS_2_-VASc score was ≥ 1 in 65 cases, CHA_2_DS_2_-VASc score ≥ 2 in 60 cases (p = 0.12 and p = 0.11 respectively in comparison with newly diagnosed AF patients).

Six months after inclusion in the study, none of the patients developed ischemic stroke or transient ischemic attack, hemorrhagic stroke (***Table 2***).

## Discussion

Our results indicate that a Federal AF screening system for patients at high cardiovascular risk by using a smartphone-case based single lead ECG which is supported by centrally located ECG specialist and central data management is feasible and reliable when performed in mixed urban and rural areas.

The sensitivity of the method varies from 94 to 98%, and the specificity is from 76 to 95% [3]. In a meta-analysis published in March 2019 by Pawel Petryszyn et al. including studies which have been published between the years 2000 – 2015 (25 studies/88786 participants), various screening methods have been compared and they did not differ significantly in efficacy except for the frequency of heart rhythm registrations. The conclusion of this meta-analysis was that the organization of the screening process is more important than technical solutions used for the AF screening [4]. However, when interpreting the presented data, it should be noted that this meta-analysis it did not include a large number of studies published after 2015, where mostly single-lead ECG monitors have been used.

According to the European recommendations and the European Heart Rhythm Association (EHRA) consensus document, the use of a single-channel ECG screening is recommended in people over 65 years with suspected asymptomatic atrial fibrillation. In this category of patients, it seems to be the most cost-effective [3]. The Ministry of Health of the Russian Federation also recommends the use of portable ECG recorders for patients with sporadic palpitations to confirm the diagnosis of atrial fibrillation or atrial flutter (recommendation class I, level of evidence B) [5].

The most significant AF screening studies are presented in the ***Table 3***. In the overwhelming majority of these studies, a portable single-lead ECG-monitor AliveCor was used.

**Table 3:** Comparative characteristics of atrial fibrillation screening studies. AF—atrial fibrillation, ECG—electrocardiography.


AUTHOR	COUNTRY, STUDY NAME	SCREENING METHOD	YEAR SCREENED	NUMBER OF MEASUREMENTS/ECG RECORDS DURATION, N	AGE ELIGIBILITY, YEARS	NUMBER SCREENED, N	NEWLY DIAGNOSED AF,%	UNINTERPRETABLE ECG,%

Hendrikx et al. [6]	Sweden	Single-lead ECG (Zenicor)	2007–2011	twice daily, during 28 days and when having palpitations/10 seconds duration	≥75	989	3.8	N/A

Proietti et al. [7]	Belgium	Single-lead ECG (Omron HCG-801)	1 week a year from 2010- 2014	1/30 seconds duration	≥18	65,747	0.47	0

Lowres et al. [8]	Australia, SEARCH-AF	Single-lead ECG (AliveCor)	2012–2013	1/no data	≥65	1,000	1.5	0.38

Kaasenbrood et al. [9]	the Netherlands	Single-lead ECG (MyDiagnostick)	2013	1/1 minute duration	≥60	3,269	1.1	0.09

Yan et al. [10]	Hong Kong	Single-lead ECG (AliveCor)	2014–2015	1/no data	≥18	13,122	0.8	0.4

Sandhu et al. [11]	Canada, PIAAF-Pharmacy	Single-lead ECG (HeartCheck, CardioComm)	2014–2015	1/30 seconds duration	≥65	1,145	2.4	1.2

Svennberg et al. [12]	Sweden, The STROKESTOP Study	Single-lead ECG (Zenicor)	2012–2014	over 2 weeks/30 seconds duration (~26.4 per subject)	75–76 year-old population	7,173	3.0	3.5

Keen et al. [13]	United States	Single-lead ECG (AliveCor)	2014–2017	1/30 seconds duration	≥65	2,286	1.6	N/A

Quinn et al. [14]	Canada, PIAAF-Family Practice	Single-lead ECG (HeartCheck, CardioComm)	2015–2016	1/no data	≥65	2,054	0.58	2,4

Soni et al. [15]	India SMART-India	Single-lead ECG (AliveCor)	2016–2017	2–3 per 5 days/2 min duration	≥50	2,074	1.6	0.05

Wang et al. [16]	China AF-CATCH	Single-lead ECG (AliveCor)	2017	1/no data	≥65	4,531	0.5	N/A

Halcox et al. [17]	United Kingdom, ECG – REHEARSE-AF Study	Single-lead ECG (AliveCor)	2017	2 per week over a 12-month period/30 seconds duration	≥65	1,004	3.8	2.2

Orchard et al. [18]	Australia, AF-SMART	Single-lead ECG (AliveCor)	2016–2018	1/no data	≥65	1,805	1.1	9.7


Our study results are comparable with results from previous studies. In particular, our results confirm previous findings that the organization of medical care is quite important. Paroxysmal atrial fibrillation cannot always be timely detected. The average waiting time for a cardiologist consultation in Russia can be to 15 days, and the duration of consultation is limited to 12 minutes. Under these circumstances, a federal cardiac monitoring system as used for this study has the potential to significantly increase the availability of medical care and the detection rate of atrial fibrillation as well as timely administration of anticoagulant therapy.

This study has several limitations: the study was conducted with a biased sample (only patients from primary care units with high cardiovascular risk were included), which does not allow to apply the results to the general population; furthermore, no cost-effectiveness analysis has been performed, which is necessary to show the full potential of this type of screening system.

In conclusion, our results indicate that a Federal AF screening system in patients at high cardiovascular risk by using a smartphone-case based single lead ECG which is supported by centrally located ECG specialist and central data management is feasible and reliable when performed in a mixed urban and rural area. Further studies are needed to evaluate the full potential of this approach.
